# Mucosal-associated invariant T cells for cancer immunotherapy

**DOI:** 10.1016/j.ymthe.2022.11.019

**Published:** 2022-12-05

**Authors:** Yan-Ruide Li, Kuangyi Zhou, Matthew Wilson, Adam Kramer, Yichen Zhu, Niels Dawson, Lili Yang

**Affiliations:** 1 Department of Microbiology, Immunology & Molecular Genetics, University of California, Los Angeles, Los Angeles, CA 90095, USA; 2 Eli and Edythe Broad Center of Regenerative Medicine and Stem Cell Research, University of California, Los Angeles, Los Angeles, CA 90095, USA; 3 Jonsson Comprehensive Cancer Center, David Geffen School of Medicine, University of California, Los Angeles, Los Angeles, CA 90095, USA; 4 Molecular Biology Institute, University of California, Los Angeles, Los Angeles, CA 90095, USA

**Keywords:** mucosal-associated invariant T cell, MAIT cell, cancer immunotherapy, allogeneic cell therapy, off-the-shelf, graft-versus-host disease, GvHD, stem cell engineering, chimeric antigen receptor engineering, CAR engineering, combination therapy

## Abstract

Human mucosal-associated invariant T (MAIT) cells are characterized by their expression of an invariant TCR α chain Vα7.2-Jα33/Jα20/Jα12 paired with a restricted TCR β chain. MAIT cells recognize microbial peptides presented by the highly conserved MHC class I-like molecule MR1 and bridge the innate and acquired immune systems to mediate augmented immune responses. Upon activation, MAIT cells rapidly proliferate, produce a variety of cytokines and cytotoxic molecules, and trigger efficient antitumor immunity. Administration of a representative MAIT cell ligand 5-OP-RU effectively activates MAIT cells and enhances their antitumor capacity. In this review, we introduce MAIT cell biology and their importance in antitumor immunity, summarize the current development of peripheral blood mononuclear cell-derived and stem cell-derived MAIT cell products for cancer treatment, and discuss the potential of genetic engineering of MAIT cells for off-the-shelf cancer immunotherapy.

## Graphical abstract

**Figure:**
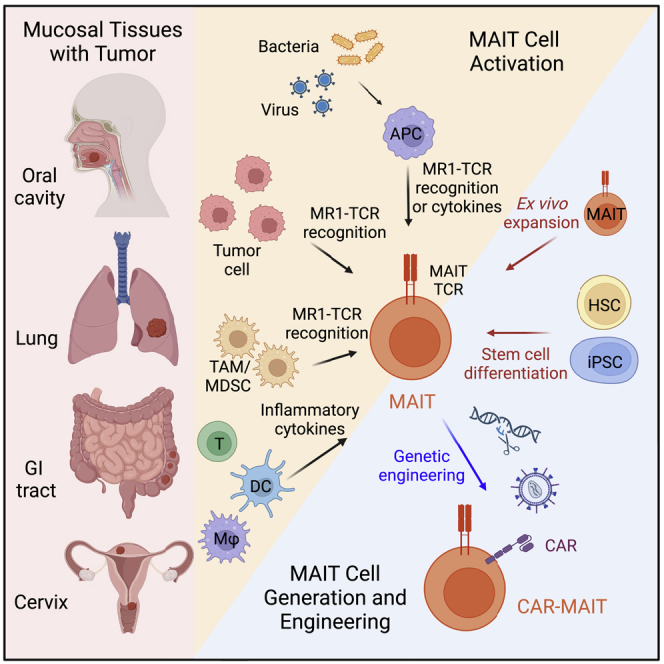

## Teaser

Human mucosal-associated invariant T (MAIT) cells are potent immune cells for targeting cancer. In this review, Li et al. summarize MAIT cell biology and their importance in antitumor reactivity, and discuss recent advances in developing PBMC- and stem cell-derived MAIT cell products for cancer immunotherapy.

## Introduction

**Figure 1:**
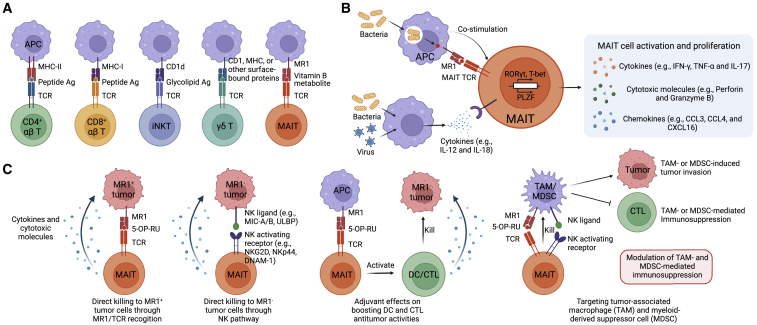
Recognition, activation, and tumor cell killing mechanism of MAIT cells (A) The main T cell subsets and their antigen recognition. Conventional αβ CD4^+^ and CD8^+^ T cells recognize peptide antigens presented through the MHC-II and MHC-I molecules on the antigen-presenting cells (APCs), respectively. The invariant natural killer T (iNKT) cells recognize glycolipid antigens presented by MHC class I-like molecule, CD1d. Gamma delta T (γδ T) cell TCR recognizes different ligands presented by diverse receptors including MHC, CD1, and other surface-bound proteins. MAIT cells express invariant αβ TCR, which binds to riboflavin (vitamin B2) biosynthesis derivatives presented by MR1 on APCs. Ag, antigen. (B) TCR-dependent and -independent MAIT cell activation. MAIT TCR-dependent activation requires riboflavin (vitamin B2) biosynthesis derivatives to be presented on MR1 to a TCR in conjunction with co-stimulation. In addition, MAIT cells can be activated by cytokines such as IL-12 and IL-18 in a TCR-independent pathway. Activated MAIT cells proliferate and secret various cytokines, chemokines, and cytotoxic molecules. (C) Tumor cell killing mechanism by MAIT cells. MAIT cells can attack tumors through multiple mechanisms, including direct killing of MR1^+^ tumor cells through MR1/TCR recognition, killing of MR1^−^ tumor cells through NK pathway, adjuvant effects on boosting dendritic cell (DC) and cytotoxic T lymphocyte (CTL) antitumor activities, and inhibition of TAMs and MDSCs.

Human mucosal-associated invariant T (MAIT) cells, a group of evolutionarily conserved, innate-like subset of T cells, currently stand as an untapped frontier with immense potential as a cutting-edge cancer immunotherapy. As their name suggests, MAIT cells primarily localize to mucosa-rich regions, comprising a notable fraction of T cells distributed throughout the pulmonary (5%), hepatic (20%–40%), and intestinal (1%–2%) lamina propria, as well as peripheral circulation (1%–10%).^1^^,^^2^^,^^3^ Given their innate-like quality, MAIT cells display a heavily restricted T cell receptor (TCR) repertoire, which has been canonically defined as expression of an invariant TCR α chain Vα7.2-Jα33 paired with a limited number of TCR β chains, predominantly Vβ2/Vβ13.^3^^,^^4^^,^^5^^,^^6^ A degree of semi-invariance has been observed in TCR α-chain recombination Vα7.2-Jα12/Jα20 within mature MAIT cells; these non-canonical MAIT subtypes display functional properties identical to those of Vα7.2-Jα33, showcasing major histocompatibility complex (MHC)-related molecule 1 (MR1)-restricted recognition, development, and cytokine profile.^7^^,^^8^ The specialized MAIT TCR specifically recognizes riboflavin-derived metabolites presented on MR1, a non-classical, MHC class I-like molecule for immunogenic cascade; cell types with high MR1 expression include bone marrow-derived antigen-presenting cells (APCs; i.e., macrophages, dendritic cells, and monocytes) and non-bone marrow-derived epithelial cells^9^^,^^10^^,^^11^ (Figure 1A). Expression of MR1 is largely localized to the ER at first, whereupon antigen loading during infection induces trafficking of MR1 to the cell surface for presentation to MAIT TCR.^12^^,^^13^^,^^14^ Activated MAIT cells showcase potent cytotoxicity, employing perforin/granzyme B to directly lyse infected cells and secreting proinflammatory cytokines (i.e., interferon-γ [IFN-γ], tumor necrosis factor α [TNF-α], interleukin-17 [IL-17], and granulocyte macrophage colony-stimulating factor [GM-CSF]) for crosstalk with neutrophils, macrophages, and other effector T cells.^2^^,^^3^^,^^15^^,^^16^

In addition to their MR1-restricted TCR, MAIT cells express a characteristic array of surface markers and receptors that contribute to their cytotoxic capacity. Agonistic engagement of Toll-like receptors (TLRs), in particular TLR1, TLR2, and TLR6, plays a role in priming MAIT TCR for activation and enhancing MAIT secretion of IFN-γ and granzyme B.^16^^,^^17^^,^^18^ MR1-dependent secretion of IL-17 induces neutrophil recruitment and upregulates secretion of proinflammatory cytokines (e.g., IL-6), chemokines (e.g., CXCL1/2/5), and antibacterial β-defensins.^19^^,^^20^^,^^21^^,^^22^ In a separate vein, MAIT cells have been shown to express an array of natural killer (NK) activating receptors (e.g., NKG2D, DNAM-1, NKp33, and NKp40), providing them with a secondary, intrinsic pathway to engage in cytotoxic effector function against MR1^−^ tumors independent of MAIT TCR.^23^ Activation along either pathway induces upregulation of CD40L for licensure of dendritic cells (DCs) to transactivate NK cells and effector T cells, propagating the immune response.^24^ Recruitment and mucosal tropism of MAIT cells is largely attributed to chemokine receptor expression; in particular, their CXCR6^+^CCR9^+^ phenotype guides trafficking toward peripheral tissues while the absence of CCR7 prevents extravasation into lymphatic structures.^2^^,^^25^ Expression of an effector memory phenotype (CD45RA^−^CD45RO^+^CD62L^low^CD161^+^) enables long-term persistence of peripheral MAIT cells until activated to mount an immunogenic response.^2^^,^^26^

The potent cytotoxic capacity and long-term *in vivo* persistence of MAIT cells grant them promise for the development of cancer immunotherapy. Importantly, MR1-restricted MAIT cells do not recognize mismatched MHC molecules and protein autoantigens; therefore, they do not induce graft-versus-host disease (GvHD).^19^ This notion is strongly supported by clinical data analyzing donor-derived MAIT cells in hematologic cancer patients who received allogeneic bone marrow or peripheral blood stem cell (PBSC) transplantation. These clinical studies demonstrated that increased levels of engrafted allogenic MAIT cells in patients correlated with improved overall survival and less GvHD incidence.^27^^,^^28^^,^^29^^,^^30^^,^^31^^,^^32^^,^^33^^,^^34^ In addition, the availability of powerful genetic engineering strategies such as lentiviral/retroviral transduction and the CRISPR-Cas9 system make it possible to genetically modify MAIT cells to enhance their antitumor reactivity (e.g., chimeric antigen receptor [CAR] engineering) or make them resistant to host immune cell-targeted depletion (e.g., ablation of MHC-I and MHC-II molecules).^23^^,^^35^ The GvHD-free safety profile and the feasibility of multiple-gene engineering position MAIT cells as ideal agents for the development of allogeneic cell therapy. However, healthy donor peripheral blood mononuclear cells (PBMCs) contain relatively low numbers of MAIT cells, making it difficult to mass produce therapeutic doses of allogeneic MAIT cells. Therefore, optimizing current protocols to efficiently expand and engineer PBMC-derived MAIT cells, or developing alternative approaches such as generating stem cell-derived MAIT cells, is of great demand.

## MAIT cell development

The early development of MAIT cells follows a pathway analogous to that of conventional T cells. Akin to MHC-mediated stimulation of αβ T cells, cortical thymic epithelial cells express MR1 to provide positive selection for MAIT TCR to stimulate MAIT cell development.^4^^,^^6^^,^^36^^,^^37^ MR1 tetramer staining has historically been used to track positive selection of MAIT cells, revealing that the progression of human MAIT maturation occurs along a three-stage pathway defined by differential expression of markers CD27 and CD161; stage 1 MAIT cells present as CD27^−^CD161^−^, transition to CD27^+^CD161^−^ in stage 2, and reach mature phenotype CD161^+^CD27^low^ in stage 3.^37^ In addition, expression of CD4 and CD8 co-receptors varies along the MAIT development pathway, with most stage 3 MAIT cells presenting either double-negative (DN) CD4^−^CD8^−^ or single-positive (SP) CD8^+^αβ phenotypes.^38^ Mature MAIT cells within peripheral circulation most closely resemble the stage 3 SP MAIT cells found in the thymus, with the exception that most mature MAIT cells transition from CD8αβ heterodimers to CD8αα homodimers after entering circulation. Despite this shift, both CD8 subtypes still maintain a similar overall phenotype, gene expression, and cytokine profile.^39^ The transition of MAIT cells from stage 2 to stage 3 is dependent on transcription factor PLZF, without which functionally mature MAIT cells will not develop; this change also upregulates expression of transcription factors RORγt and T-bet, arming mature MAIT cells with IL-17 and IFN-γ secretion for immunogenicity and crosstalk upon thymic exfiltration.^37^^,^^38^^,^^40^

## MAIT cell activation

### TCR-dependent activation

As previously mentioned, MAIT cells are primarily activated by TCR-dependent recognition of MR1-presented antigens (Figure 1B).^41^ Expression of MR1 on APCs includes dendritic cells, epithelial cells, macrophages, and monocytes.^42^^,^^43^^,^^44^ MR1 is a highly conserved across mammalian species and presents riboflavin (vitamin B2) intermediates to MAIT TCRs.^45^^,^^46^ MR1 can bind to and stabilize otherwise unavailable intermediate structures to present to MAIT cells, heightening the sensitivity of MAIT-mediated immunosurveillance of microbial signatures.^48^

In particular, MAIT TCR recognizes the vitamin B2 precursors 5-(2-oxopropylideneamino)-6-D-ribitylaminouracil (5-OP-RU) and 5-(2-oxoethylideneamino)-6-D-ribitylaminouracil (5-OE-RU) presented on MR1.^17^^,^^41^ The metabolite 5-amino-6-ribitylamino-2,4-(1H,3H)-pyrimidinedione (5-A-RU), a key intermediate in the synthesis of riboflavin, has also been found to be particularly important in the activation of MAIT cells.^49^ When 5-A-RU reacts with methylglyoxal or glyoxal, pyrimidine adducts 5-OP-RU or 5-OE-RU can be synthesized, respectively.^46^^,^^49^ Described over a decade ago by Le Bourhis et al., organisms that utilize the riboflavin biosynthetic pathway include several strains of bacteria and yeast, notably excluding viruses.^11^^,^^47^ Consistent with this finding, absence of this pathway precludes MR1-dependent activation of MAIT cells.^47^

Co-stimulation is vitally important for the activation of MAIT cells, as stimulation by MR1 alone is insufficient to elicit a cytotoxic response. Even with repeated administration of 5-OP-RU, MAIT cells are unable to accumulate within inoculated tissues *in vivo*, requiring additional stimulatory signals from infected APCs.^17^ Consistently, co-stimulation with CD28 and innate cytokines such as IL-18, IL-23, and IL-1β dramatically increases MAIT cell proliferation and activation.^50^ Current research aims to utilize MAIT cell activation as an adjuvant toward other therapies and vaccines. MAIT activation shows support for the immunogenicity of drug and vaccine candidates, and regulates NK cell-mediated antitumor cytotoxic activity *in vivo*, potentially attributed to an enhanced type-I IFN response that occurs upon activation.^51^^,^^52^ Thus, combination therapies targeted toward the activation of MAIT cells may serve as a viable treatment option in the future.

### TCR-independent activation

Although MAIT cell activation is mainly instigated through MR1/TCR interactions, MAIT cells can be partially activated through cytokine stimulation pathways^41^ (Figure 1B). Most notably, IL-12 and IL-18 have been well studied in TCR-independent activation of MAIT cells;^15^^,^^53^ in addition, stimulation from IL-7, IL-15, and type-I IFNs may also contribute toward TCR-independent MAIT cell activation.^43^ It has been proposed that cytokine activation of MAIT cells provides an additional defense against viral infections, considering that infected cells do not produce riboflavins for MR-1-mediated activation.^43^ For the most part these activation pathways are stimulated through TLR signaling, as well as upregulation of the IκBξ transcription factor, to enhance IFN-γ secretion.^15^^,^^43^^,^^53^ The mechanisms behind IL-12 and IL-18 activation remains unclear at this point; however, ongoing research continues to investigate the intricacies of this pathway in the context of hepatitis C and autoimmune disease.^53^ Current studies in MAIT cell activation may elucidate mechanisms to improve current cell-based therapies or help develop novel approaches for the management of bacterial infections, viral diseases, or cancerous malignancies through mechanistic discoveries of the cytotoxic and synergistic qualities of MAIT cells.

## MAIT cells in cancer

Since their discovery, MAIT cells have attracted increasing attention regarding their application in the context of tumor immunology.^37^^,^^54^ In a meta-analysis of expression signatures from diverse tumor samples, expression of *KLRB1* (encoding CD161) by tumor-infiltrating leukocytes was identified as the most favorable prognosis marker across 39 malignancies.^55^ Although expression of CD161 is shared with activated cytotoxic CD8 T cells and NK cells,^56^ MAIT cells constitute a major proportion of CD161^+^ infiltrating T cells in the periphery, evident through the predominance of TCR Vα7.2 co-expression in tumor-infiltrating lymphocytes.^55^ Thus, it is tempting to speculate that MAIT cells may play a critical role in tumor immunity, especially for solid tumors, although the exact molecular mechanisms involved have yet to be clarified. However, current clinical evidence detailing the role of MAIT cells in cancer prognosis is dichotomous, as both pro- and antitumor characteristics of MAIT cells have been observed within both solid tumors and hematologic malignancies.^52^^,^^57^^,^^58^^,^^59^^,^^60^^,^^61^^,^^62^^,^^63^^,^^64^^,^^65^^,^^66^

MAIT cell tropism toward mucosal-associated peripheral tissues within the lungs, gastrointestinal tract, colon, and cervix inextricably implicate MAIT with tumor prognosis within the lamina.^58^^,^^61^^,^^62^^,^^63^^,^^64^^,^^66^ Several studies specifically focusing on MAIT cells in colorectal cancer (CRC) have observed a reduction of MAIT cell abundance in the periphery due to preferential migration toward neoplastic sites.^61^^,^^63^ Similar changes in MAIT biodistribution were observed in gastric cancer (GC) and cervical cancer patients as well.^64^^,^^66^ In contrast, lung cancer patients exhibited an elevated proportion of circulating MAIT cells compared with healthy individuals.^58^ A study employing an *in vivo* lung cancer mouse model determined that tumor initiation, growth, and metastasis were significantly reduced for MR1 knockout mice, suggesting that MAIT cells promote tumor progression in a TCR-MR1-dependent manner.^59^ This is consistent with observations in tumor-infiltrating MAIT cells in CRC and GC patients, where exhausted MAIT cells (PD-1^high^Tim-3^+^CD39^+^) became the dominant phenotype and exhibited greater penetration into CRC tissues,^61^ and MAIT cells showed reduced secretion of granzyme B molecule.^64^ However, a study of colon adenocarcinoma reports contradictory results, wherein MAIT cells that infiltrated the tumor site instead had unchanged cytotoxic potential with normal expression of granzyme B and CD107a.^65^ Despite the discrepancy in MAIT distribution between different tumor types, tumor-localized MAIT cell accumulation seems to produce adverse outcomes for patients on account of an exhausted, immunosuppressive phenotype.

In addition to mucosal-associated tissues, distribution of MAIT cells is also enriched within the liver and in peripheral circulation, implicating involvement of MAIT cells in related cancers. In patients with hepatocellular carcinoma (HCC), neoplasm-localized MAIT cells exhibited upregulation of inhibitory immune molecules (e.g., PD-1, CTLA-4, and TIM-3) and secreted lower quantities of effector molecules (e.g., IFN-γ, granzyme B, and perforin).^4^ As suggested by this immunosuppressive phenotype, high MAIT cell infiltration into HCC solid tumors was correlated with adverse prognosis.^60^ With regard to non-solid, hematologic cancers, the implications of the presence of MAIT have not been explored as extensively, although some preliminary studies have been conducted. In a study of multiple myeloma (MM), peripheral blood samples from patients indicated a significantly reduced abundance of MAIT cells, and remaining cells suggested suppression of antitumor capacity through depressed secretion of IFN-γ and TNF-α and elevated expression of PD-1.^67^^,^^68^^,^^69^ The discrepancy between the protumoral effects of MAIT cells within the tumor microenvironment and their cytotoxic potential suggests a manipulable plasticity that is exploited through extrinsic tumor signaling. Thus, MAIT cells may be considered as a promising target for immunotherapy, either through reprogramming their protumor phenotype through pharmacological interference or genetically engineering MAIT cells toward an antitumor state.^70^

There is emerging evidence suggesting that the human microbiota is associated with cancer in various ways, especially within the solid tumor microenvironment.^71^^,^^72^^,^^73^^,^^74^^,^^75^ Certain variations in the microbial composition and/or microbial signatures serve as prognostic markers and are known to promote tumor initiation, growth, or metastasis.^73^^,^^74^^,^^75^ On the other hand, there are studies showing that the microbiota might also play a regulatory role or enhance the efficacy of cancer immunotherapy.^76^^,^^77^^,^^78^ Given the tissue tropism of MAIT cells toward microbe-enriched regions *in vivo* and their ability to respond to MR1-presented microbial metabolites, such as riboflavin derivatives, MAIT cells are likely to participate in the interplay between the human microbiome and the solid tumor microenvironment. It has been reported that the basal level expression of MR1 on a variety of tumor cell lines is low to undetectable; however, upon exposure to microbial metabolites 5-OP-RU, B16F10 melanoma cells significantly upregulated MR1 surface expression in a dose-dependent manner, but not for RIL-175 or CT269. Furthermore, knockout of MR1 in B16F10 did not affect the antitumor capacity of activated MAIT cells *in vivo*.^79^ Taken together, these results suggest that although MAIT cells exhibit antitumor response upon activation by microbial metabolites 5-OP-RU, the tumor killing is not dependent on the tumor MR1 expression. While further efforts are needed to confirm the relevance of the MAIT TCR/MR1/5-OP-RU axis in the direct cytotoxicity of MAIT cells against tumors, molecular mechanisms that contribute to the discrepancy of MR1 upregulation between different tumor cell lines require further investigations.

In addition to the important roles of proinflammatory type-1 MAIT cells toward combating against microbial infections, type-17 MAIT cells (RORγt^+^Tbet^–^), which secrete IL-17A, exhibit vital functions associated with tissue homeostasis and repair, particularly within mucosal-associated areas.^41^^,^^80^^,^^81^^,^^82^ Previous studies have summarized the latest advances in dissecting the developmental trajectories and specific environmental cues that might skew MAIT cells toward tissue-repair-related phenotypes within both human and murine models.^83^^,^^84^ In human, TCR-dependent activation induced enrichment of tissue-repair-associated type-17 MAIT cells, while TCR-independent activation mainly promoted antimicrobial inflammatory type-1 MAIT cells, secreting IFN-γ and TNF-α.^82^ Although there is a paucity of murine MAIT cells present in peripheral blood, Constantinides et al. showed that MAIT cells were significantly enriched within murine skin and were predominantly of type-17 phenotype.^80^ While the model of bidirectional polarization of type-1 and type-17 MAIT cells has been established, little is known about whether or how type-17 tissue repair might play a role in tumor immunity. This might be partially explained by the issue that current functional studies and transcriptional analysis of type-17 MAIT cells have failed to elucidate the exact molecular mechanism by which type-17 MAIT cells contribute to tissue repair.^84^ It is conceivable to postulate that the seemingly immunosuppressive phenotype of type-17 MAIT cells might play a protumor role in the solid tumor microenvironment, and previous evidence has shown that the closely related type-17 γδ T cells contribute to tumor growth and metastasis in human cancer by secretion of IL-17A, IL-8, and GM-CSF, which in turn recruit myeloid-derived suppressor cells (MDSCs).^85^ However, whether type-17 MAIT cells are indeed immunosuppressive and protumor or if a similar cellular crosstalk exists between MDSCs and type-17 MAIT cells has yet to be determined. Additional questions revolving around the role of type-17 MAIT cells in malignancies include whether the polarization of type-17 MAIT cells can be manipulated using defined cytokine cocktails *ex vivo* or whether genetic engineering such as the construction of CARs can be used to direct MAIT cell polarization into an antitumor phenotype.

## Peripheral blood mononuclear cell-derived MAIT cells for cancer immunotherapy

### Current protocols to culture PBMC-MAIT cells

**Figure 2:**
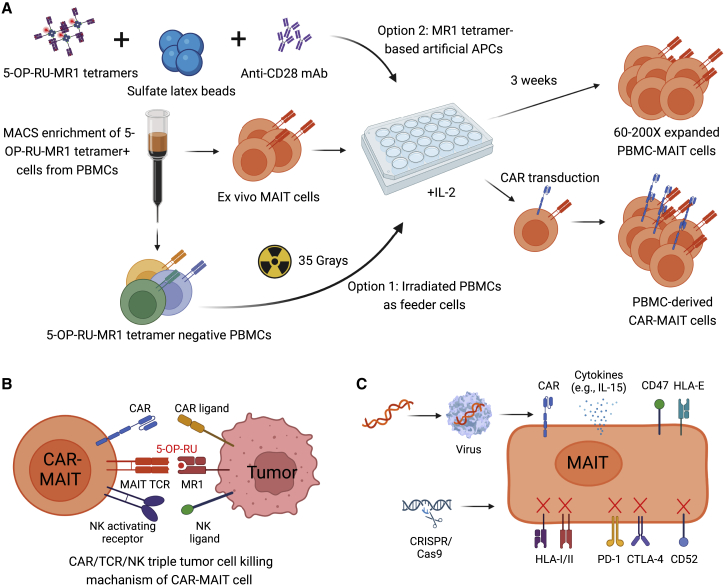
Generation and genetic engineering of human peripheral blood mononuclear cell-derived MAIT (PBMC-MAIT) cells (A) Generation of MAIT or CAR-engineered MAIT (CAR-MAIT) cells from human PBMCs. MACS-enriched *ex vivo* MAIT cells can be co-cultured with either irradiated MR1-tetramer-negative PBMCs or latex bead-based artificial APCs. (B) CAR/TCR/NK triple tumor cell killing mechanism. CAR-MAIT cells target tumor cells through CAR ligand/CAR, MR1/5-OP-RU/TCR, and NK ligands/NK activating receptors. The multiple tumor targeting mechanisms grant MAIT cells a stronger antitumor capacity and an enhanced capacity to counteract tumor antigen escape. (C) Multiple genetic engineering approaches could be incorporated into the generation of PBMC-MAIT cells, such as arming CARs to enhance antitumor efficacy, overexpressing cytokines (i.e., IL-2 and IL-15) to boost immune reaction, ablating HLA-I and HLA-II to reduce host T cell-mediated alloresponse, transducing HLA-E or CD47 to reduce host NK cell-mediated alloresponse, knocking out checkpoints (i.e., PD-1 and CTLA-4) to reduce the immunosuppression, and depleting CD52 to grant cells resistance to the T cell depletion preconditioning treatment.

Existing protocols, using either MR1-tetramer-based artificial APCs^86^ or 5-OP-RU-loaded irradiated PBMCs as feeder cells,^87^ to culture and expand healthy donor PBMC-sorted MAIT cells (defined as MR1-tetramer^+^CD161^hi^Va7.2^+^CD3^+^CD8^+^) *ex vivo*, showed limited expansion fold change ranging from 60- to 200-fold.^86^^,^^87^ Since the proposal of MAIT cells for cancer immunotherapy was raised,^16^ this seemingly hypoproliferative nature of PBMC-MAIT cells has become a major bottleneck that hinders any downstream applications of PBMC-MAIT cells for preclinical evaluation and translation.^2^ The long-lasting technical difficulty can be partially explained by the unexpectedly poor proliferation of MAIT cells cultured *in vitro* with a supply of conventional T cell expansion cytokine cocktail upon TCR-dependent activation or mitogen stimulation.^2^^,^^86^^,^^87^ It is unlikely that the hypoproliferation of *ex vivo* cultured PBMC-MAIT cells is intrinsic, as evidence exists in both mouse and human studies suggesting that MAIT cells expand robustly *in vivo* upon activation.^59^^,^^79^ Thus, further investigations are required to understand the mechanisms, on both cellular and molecular levels, by which PBMC-MAIT cells expand differently from conventional PBMC-T cells *ex vivo*. In this section, we review and compare the latest protocols used to expand PBMC-MAIT cells *ex vivo* and discuss current evidence that might facilitate further optimization of PBMC-MAIT cell culture methods (Figure 2A).

In 2021, Parrot et al. reported development of an *ex vivo* expansion protocol for PBMC-sorted human MAIT cells, combing immunomagnetic bead-based cell sorting and 5-OP-RU-loaded irradiated PBMC feeder cells.^87^ Starting from the peripheral blood of healthy donors, human MAIT cells are enriched by magnetic activated cell sorting (MACS) based on positive selection using 5-OP-RU-loaded MR1-tetramers.^87^ On the same day, the negative portion of PBMCs after MAIT cell depletion is irradiated and co-cultured with MACS-enriched MAIT cells in a 10:1 ratio.^87^ When investigating cytokine influence on MAIT proliferation, Parrot et al. used IL-2, IL-7, and IL-15 at different concentrations in expanding MAIT cells *ex vivo*. In their hands, IL-2 or IL-15 alone induced better expansion of MAIT cells in comparison with IL-7 alone (1–100 ng/mL tested).^87^ The activation and expansion of MAIT cells observed is likely due to a combinatorial effect of cytokines and 5-OP-RU-loaded MR1-tetramer MACS sorting, which provide TCR-dependent activation, and co-stimulatory signals from the negative portion of PBMCs containing APCs. The overall 200-fold *ex vivo* expansion of PBMC-MAIT cells reported in this study is among the highest in current literature; additionally, usage of antiCD3/CD28/CD2 stimulation did not further increase the yield but resulted in increased conventional T cell impurity by day 20,^87^ likely explained by imperfection of MACS efficiency whereby conventional T cells routinely compose around 5% of CD3^+^ T cells after MR1-tetramer MACS sorting.^86^^,^^87^ The more robust TCR-dependent activation of conventional T cells rendered more potent expansion compared with MAIT cells, which in turn compromised MAIT cell percentage in final products.^88^ As suggested by Slichter et al. in 2016, TCR-dependent activation of conventional CD8^+^ T cells using anti-CD3/CD28 beads induced significantly more prolonged activation, indicated by elevated production of IFN-γ and TNF-α (monitored up to 24 h); in comparison, MAIT cells demonstrated a transient peak expression at 6 h, which had rapidly been lost by 12 h.^88^ Addition of IL-12, IL-15, and IL-18 resulted in a significantly increased proportion of IFN-γ^+^ and granzyme B^+^ MAIT cells, but not for conventional CD8^+^ T cells.^88^ These results suggest distinct TCR-dependent activation models between MAIT cells and conventional CD8^+^ T cells, whereby MAIT cells require a synergistic mixture of both TCR signaling and inflammatory cytokines, such as IL-12 and IL-18, to achieve complete activation.^88^ The importance of inflammatory cytokines for activating MAIT cells is also supported by the data from Parrot et al., where co-culture of MAIT cells with irradiated PBMCs resulted in over 20-fold increase of MAIT cell expansion, in comparison with culturing with IL-2 or IL-15 alone.^87^ However, specific proinflammatory cytokines produced by APCs or other cell sources that contribute to the *ex vivo* expansion of MAIT cells remain unclear. Slichter et al. previously showed that TLR4/TLR8 agonist-treated monocytes did not produce IL-12 or IL-18 but still induced activation of MAIT cells upon co-culturing.^88^ This suggests that additional inflammatory signals might be involved to direct MAIT cell activation toward an effector-like state,^88^ although the exact signal milieu requires further investigation. While previous studies have demonstrated that IL-12 and IL-18 are sufficient to activate MAIT cells even in the absence of TCR activation,^88^ it remains elusive whether or how these inflammatory cytokines might contribute to *ex vivo* expansion of MAIT cells.

Priya and Brutkiewicz reported an alternative protocol which utilizes MR1 tetramer-based artificial APCs to activate MAIT cells.^86^ In brief, cell-sized latex beads are coated with 5-OP-RU-loaded MR1 tetramers and anti-CD28 antibodies overnight.^86^ When supplied with IL-2, MAIT cell expansion reached around 74-fold.^86^ While there are clinical advantages of utilizing latex-bead-based artificial APCs over a feeder-based system, such as a lower risk of introducing cell culture contamination or convenience for quality control, any discrepancies between feeder-cell-based and latex-bead-based MAIT cell expansion fold change needs to be further investigated. One possible direction is to elucidate whether a difference in the surface density of MR1 molecules present on natural PBMCs or artificial APCs might affect the final expansion fold change of MAIT cells *ex vivo*. Substantial evidence exists showing that MR1 expression on most cell types is at low to undetectable levels under normal conditions, and upregulated upon exposure to MAIT cell antigens such as 5-OP-RU.^16^^,^^79^ It is thus reasonable to postulate that a dynamic regulation of MR1 serves as a critical checkpoint for MAIT cell activation. More insight into how and why MAIT cell activating ligands regulate MR1 surface expression will not only benefit further optimization of *ex vivo* culture of MAIT cells but also provide guidance for developing MAIT cell-based immunotherapy.

### The antitumor effector and regulatory properties of PBMC-MAIT cells

Although there is considerable evidence suggesting a role of MAIT cells in both solid and hematologic cancer, the exact functions of MAIT cells and their crosstalk with other immune cells in the context of malignancies remain largely unknown. While there is a common observation that circulating MAIT cells preferentially infiltrate into peripheral tumor microenvironments^61^^,^^62^^,^^63^^,^^64^ and exhibit an exhausted phenotype with compromised effector molecule production,^60^ emerging evidence suggests that either anti-CD3/CD28 stimulation or addition of inflammatory cytokines such as IL-12 and IL-18 can rescue this exhausted phenotype to a certain extent.^65^ Notably, circulating and tissue-resident MAIT cells respond differently upon stimulation.^65^ A combination of inflammatory cytokines IL-12 and IL-18 induced increased production of granzyme B in both circulating and tissue-resident MAIT cells.^65^ However, TCR stimulation through anti-CD3/CD28 only induced potent granzyme B production in tissue-resident MAIT cells.^65^ This discrepancy of cytotoxic potential of MAIT cell subtypes upon TCR stimulation might partially explain the opposing pro- or antitumor MAIT cell phenotypes found in different studies related to a same type of cancer.^61^^,^^62^^,^^63^^,^^64^ However, since there is no consensus on how circulating or tissue-resident MAIT cells are defined, clinical analysis of the role of MAIT cells in either suppressing or supporting malignant cell growth remains ambiguous. It is thus of importance to utilize relevant mouse models, such as MR1 knockout, to elucidate specific molecular pathways that contribute to a pro- or antitumoral function in MAIT cells.^59^

*Ex vivo* expanded MAIT cells exhibit *in vitro* killing capacity against a variety of tumor cell lines^69^ (Figure 1C). MAIT cells targeted and lysed 5-OP-RU-pulsed MM cell lines (RPMI-8226 and U266) in an MR1-dependent manner, indicated by complete abrogation of effective MAIT cell killing with addition of anti-MR1.^69^ However, it is likely that MAIT cells are able to target tumor cells in a TCR-MR1 axis-independent manner, since *ex vivo* cultured MAIT cells not only suppressed MR1^+^ tumor cell lines *in vitro* but also eliminated MR1^−^ tumor cells (unpublished data). MAIT cells express activating NK receptors such as NKG2D and DNAM-1, which may in turn recognize NK-related stress ligands (MIC-A/B and/or ULBP-1) present on tumor cells and confer NK-mediated cytotoxicity; however, the exact mechanisms require further validation. Furthermore, consistent results exist in several *in vivo* studies.^79^ The *in vivo* antitumor capacity of MAIT cells has been evaluated in several mouse models such as HCC, lung metastasis, and subcutaneous tumors.^79^ Specifically, a combined treatment of 5-OP-RU and TLR9 agonist CpG induced robust *in vivo* expansion of MAIT cells with high CD69 expression.^79^ Activated MAIT cells secreted high levels of effector molecules such as IFN-γ, granzyme B, and perforin, which in turn led to a significant tumor-suppressing immune response.^79^ Interestingly, tumor targeting by MAIT cells *in vivo* seemed not to be completely dependent on MR1 expression on tumor cells, since MR1 knockout had only minor effect on MAIT cell antitumor response.^79^ In addition to the aforementioned NK-mediated killing pathway of MAIT cells, it is equally possible that in an *in vivo* system, MAIT cell activation might be alternatively achieved through environmental cues such as inflammatory cytokines induced by TLR9 agonist CpG.

While MAIT cells exhibit direct antitumor cytotoxicity upon activation,^69^^,^^79^^,^^89^ recent studies also suggest an immune-regulatory role of MAIT cells in tumor immunity.^52^ Evidence from MAIT cell-deficient mouse models showed resistance to B16F10 lung metastasis and subcutaneous tumor growth.^52^ Mechanistically, the group proposed an MAIT-NK cell axis, where MAIT cells at steady state negatively affect NK cell maturation and activation, as was evident by MAIT cell-deficient mice displaying an increased number of NK cells; however, this protective effect was abrogated when NK cells were depleted.^52^ More importantly, the suppressive role of quiescent MAIT cells on NK-mediated antitumor immunity was completely reversed when MAIT cells were activated *in vivo* by 5-OP-RU.^52^ Activated MAIT cells showed increased expression of IFN-γ and induced robust NK cell activation and expansion, which in turn promoted significant antitumor immunity.^52^ The IFN-γ-dependent activation of NK cells by MAIT cells is reminiscent of a regulatory effect provided by activated NK T cells, reported by several other studies.^90^^,^^91^ Similarly, recruitment of CD8^+^ cytotoxic conventional T cells to the tumor microenvironment seemed to be mediated by IFN-γ producing MAIT cells as well,^65^ although follow-up studies are required to fully elucidate the underlining interplay between different immune networks. Overall, it has become clear that MAIT cells constitute a heterogeneous population of distinct subtypes, which are programmed by the specific microenvironment in which they reside. Although MAIT cells are subject to polarization toward an exhausted phenotype within the immunosuppressive tumor microenvironment,^61^^,^^63^ MAIT cells exhibit significant plasticity under extrinsic stimulations.^65^

## Stem cell-engineered MAIT cells for cancer immunotherapy

### Methodology and current progress in HSC-engineered MAIT cells

**Figure 3:**
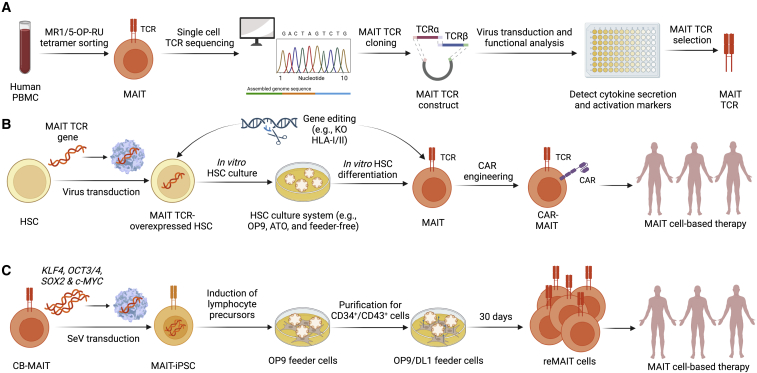
Generation and genetic engineering of human stem cell-derived MAIT cells (A) Cloning of human MAIT TCR genes. Single human MAIT cells are sorted from healthy donor PBMCs using flow cytometry. The sorted single MAIT cells are subjected to TCR cloning using a single-cell TCR sequencing and cloning technology. A pair of MAIT TCR α- and β-chain genes are selected based on the functional analysis such as cytokine secretion capacity and activation marker expression. (B) Generating MAIT cells from hematopoietic stem cells (HSCs). Human CD34^+^ HSCs from cord blood or peripheral blood stem cells are transduced with MAIT TCR and then cultured in an *in vitro* HSC differentiation system, such as OP9-DL1, artificial thymic organoid (ATO), or feeder-free culture. At the end of culture, MAIT cells with transduced MAIT TCR are generated. MAIT cells can be engineered with CARs to enhance their antitumor capacity. Gene editing such as CRISPR-Cas9 can be incorporated into HSCs or MAIT cells to achieve specific gene modifications. (C) Generating MAIT cells from induced pluripotent stem cells (iPSCs). Cord blood MAIT cells are transduced with Sendai viral vectors carrying KLF4, OCT3/4, SOX2, and c-MYC. MAIT-iPSC cell lines are established after passing the standardized pluripotency tests. MAIT-iPSCs are cultured on OP9 feeder cells and differentiate into CD34^+^/CD43^+^ lymphocyte precursors. CD34^+^/CD43^+^ precursors are then cultured on OP9/DL1 feeder cell layers for 30 days to form MAIT-iPSC-derived MAIT-like (reMAIT) cells.

Hematopoietic stem cells (HSCs) have been used for developing therapeutic cells, especially allogeneic immune cells. Multiple off-the-shelf cell culture systems, such as OP9-DL, artificial thymic organoid, and feeder-free culture systems, were utilized to support *in vitro* HSC differentiation and mature immune cell generation.^92^^,^^93^ By combining TCR gene engineering on HSCs and *in vitro* differentiation, TCR-transgenic T cells could be efficiently generated to specifically target tumor antigens (Figures 3A and 3B). Because of allelic exclusion, these TCR-redirected T cells do not rearrange endogenous TCR loci and express only the transgenic TCR, resulting in a diminished risk of GvHD.^94^^,^^95^^,^^96^

Various TCRs have been applied to HSC engineering, including NY-ESO-1 TCR, MART1 TCR, and iNKT TCR.^35^^,^^97^^,^^98^^,^^99^^,^^100^^,^^101^ Our previous works have demonstrated the successful generation of autologous and allogeneic HSC-derived iNKT cells for cancer immunotherapy.^35^^,^^101^ Human cord blood (CB) or PBSC CD34^+^ HSCs were transduced with a lentivector encoding a human iNKT TCR gene and/or a suicide gene, followed by a streamlined 6- to 10-week culture to differentiate into human iNKT cells. These generated HSC-engineered iNKT cells closely resembled human endogenous iNKT cells, effectively targeted tumor cells, and exhibited high safety and low immunogenicity.^35^ The HSC-derived iNKT cell therapy showed promise in the treatment of a variety of cancers, including hematologic malignancies and solid tumors.^35^ Notably, the same strategy could be readily utilized to generate HSC-derived MAIT (HSC-MAIT) cells by transducing MAIT TCR into HSCs and culturing these HSCs in our established off-the-shelf HSC differentiation platform. The HSC-MAIT cell platform is robust and versatile, allowing the plug-in of additional engineering approaches. In previous studies, we achieved high efficacy of CAR engineering and HLA knockout on HSC-derived immune cells through retroviral transduction and CRISPR-Cas9 gene editing, respectively.^35^ Additionally, other genetic engineering approaches, such as overexpression of immune-enhanced genes (i.e., IL-15 and IL-2) and ablation of checkpoints (i.e., PD-1 and CTLA-4), could be incorporated into the proposed HSC-MAIT cell products, paving the way for harnessing HSC-MAIT cell translational potential in universal off-the-shelf cancer immunotherapy (Figures 2C and 3B).

### Methodology and current progress in PSC-engineered MAIT cells

Owing to the suboptimal *ex vivo* expansion efficiency of MAIT cells from healthy donor peripheral blood, much effort has been invested early on to generate pluripotent stem cell (PSC)-derived MAIT-like cells^102^ (Figure 3C). In pioneering work by Wakao et al. in 2013, CB MAIT cells were reprogrammed into induced PSCs (iPSCs) using a Sendai viral vector encoding KLF4, OCT3/4, SOX2, and c-MYC.^102^ The resulting MAIT-iPSC clones passed multiple pluripotency tests, including telomerase activity, OCT3/4 and NANOG promoter demethylation, expression of pluripotency-related transcripts, *in vitro* differentiation into all three germ layers, and *in vivo* formation of teratomas in immunocompromised mice.^102^ To differentiate established MAIT-iPSC clones into MAIT-like lymphocytes, a two-step protocol was developed whereby MAIT-iPSCs were first induced on feeder cells OP9, generating lymphoid lineage precursors defined as CD34^+^CD43^+^. On day 11, differentiated cells were purified and seeded onto OP9/DL1, allowing T cell differentiation. After another 4-week culture on OP9/DL1, MAIT-like cells were stimulated with anti-CD3/CD28 magnetic beads.^102^ These MAIT-iPSC-derived MAIT-like cells were denoted as reMAIT cells by the authors, and this term is thus used hereafter. Although over 98% of the final products are Vα7.2^+^IL-18Rα^+^CD161^hi^, a high percentage of reMAIT cells (∼89%) exhibit CD45RA^+^CD4^+^, in contrast to the dominant phenotype CD45RO^+^CD8^+^ in PBMC-MAIT cells.^102^ Moreover, reMAIT cells expressed low levels of CD25, CD27, CD28, NKG2D, NKp80, and IL-2R, indicating a relatively naive state in comparison with PBMC-MAIT cells. Intriguingly, when reMAIT cells were adoptively transferred into immunocompromised mice, memory markers and/or T cell homing receptors changed dramatically. For instance, over 90% of reMAIT cells that reside intraepithelially became CD45RO^+^ and were moderately high for CCR5 and CCR6, suggesting an effector memory state and increased peripheral infiltrating capacity.^102^ These results indicate that there is a lack of environment cues provided by the current reMAIT culture protocol, and additional cytokines and/or supporting conditions are needed to push reMAIT cells into a fully activated and effector state that resembles endogenous PBMC-MAIT cells. Nonetheless, these reMAIT cells have been found to localize to different organs in mice during maturation.^89^ Following tumor inoculation, reprogrammed MAIT cells inhibited tumor growth and increased survival in the lung metastasis mouse model.^35^ The technology thus provides great opportunities to translate iPSC-derived MAIT-like cells for cancer immunotherapy; however, more work is required to fully elucidate the intrinsic differences between reMAIT cells and PBMC-MAIT cells, both *in vitro* and in mouse models.

The Wakao group tested the clinical relevance of adoptively transferred murine iPSC-derived MAIT cells in suppressing Lewis lung carcinoma (LLC) in C57BL/6 (Ly5.1) mice.^102^ Adoptive transfer of mouse reMAIT cells significantly increased survival of mice intravenously inoculated with LLCs, but failed to suppress tumor growth at any given dosage upon *in situ* subcutaneous LLC inoculation.^102^ While this study provides valuable insights into adoptive transfer of MAIT-like cells targeting solid tumors, critical limitations should be taken into consideration. The phenotypic and functional characteristics of reprogrammed MAIT cells do not fully recapitulate those of PBMC-derived MAIT cells, and, more importantly, homing chemokine receptors expressed on reMAIT cells exhibit a striking difference compared with those detected on endogenous PBMC-MAIT cells, suggesting developmental immaturities. In addition, there is a lack of direct experimental readouts for evaluating the solid-tumor-infiltrating capacity of reMAIT cells. Although the data suggest that adoptively transferred reMAIT cells are not sufficient to suppress solid tumor *in situ*, whether there is infiltration of MAIT cells inside solid tumor mass remains unclear. It is equally plausible that reMAIT cells are able to infiltrate but become exhausted within the immunosuppressive tumor microenvironment, reminiscent of what is observed in clinical samples. Given the low efficacy of conventional CAR-engineered T cells in targeting solid tumors, it is tempting to develop innate-like T cell-based (such as MAIT cells which exhibit intrinsic peripheral infiltrating capacity) cancer immunotherapy for solid tumors.

## Chimeric antigen receptor-engineered MAIT cells

Because of the differential expression of several antigens (e.g., CD19, BCMA, GD2, GPC3, and mesothelin) on certain cancer cells, CAR-engineered T (CAR-T) cells specific for these antigens have exhibited great potential in cancer immunotherapies.^103^^,^^104^ However, owing to the high cost of current and upcoming CAR-T cell therapies, affordability blocks access to the majority of patients.^105^ Current CAR-T therapies are autologous, limiting the potential to generate universal off-the-shelf products. Therefore, researchers have turned to innate lymphocytes as possible directions for a new CAR product (Table 1).^106^ Expressing highly conserved TCR profiles and independent from classical MHC recognition, innate T cells are ideal targets for CAR engineering and potentially demonstrate more favorable antitumor immunity and safety profile, displaying nearly no graft-versus-host alloreaction and limited off-target cytotoxicity and cytokine release syndrome (CRS).^35^^,^^106^^,^^107^^,^^108^ CD19, BCMA, and GD2 CAR-engineered iNKT cells, and GD2 and GPC3 CAR-engineered γδ T cells, were developed with enhanced short- and long-term antitumor activity, the ability to maintain CAR-dependent and -independent cytotoxicity, and better safety profiles compared with their CAR-T counterparts.^35^^,^^109^^,^^110^^,^^111^^,^^112^ Based on these findings, innate T cells hold great potential as a platform for allogeneic immunotherapy development.

**Table 1:** Comparison of biology and antitumor reactivity between three innate-like T cells

Innate T cell type	MAIT cells	iNKT cells	γδ T cells
TCR αβ chain	α chain: Vα7.2-Jα33 β chain: Mainly Vβ2 and Vβ13	α chain: Vα24-Jα18 β chain: dominantly Vβ11	–
TCR γδ chain	–	–	γ chain: Vγ2, Vγ3, Vγ4, Vγ5, Vγ8, Vγ9, and Vγ11 δ chain: mainly Vδ1, Vδ2, and Vδ3
MHC restriction	MR1	CD1d	CD1d, butyrophilins-3A (CD277)
Antigen recognition	intermediates of riboflavin biosynthesis (e.g., 5-OP-RU)	glycolipid antigens (e.g., α-GalCer)	phosphoantigens from microbials (e.g., HMBPP); metabolites from the mevalonate pathway (e.g., IPP)
Identification by flow cytometry	CD3^+^ TCR αβ^+^ TCR Vα7.2^+^ CD161^+^ MR1/5-OP-RU tetramer^+^	CD3^+^ TCR αβ^+^ TCR Vα24^+^ TCR Vβ11^+^ CD1d/α-GalCer tetramer^+^	CD3^+^ TCR γδ^+^
NK feature	NKG2D, DNAM-1, NKp33, and NKp40 high	NKG2D, DNAM-1, CD161, NKp33, and NKp40 high	NKG2D, DNAM-1, NKp30, NKp44, and NKp46 high
GvHD risk	low	low	low
Resident organs	lung, gastrointestinal tract, colon, and cervix	liver, lung, adipose tissue, and intestine	skin, intestine, and lung
Abundance in blood	around 1%–10%	around 0.001%–1%	around 1%–10%
Application in cancer therapy (with references or NCT number)	ovarian cancer (mesothelin-targeting CAR-MAIT^23^) breast cancer (Her2-targeting CAR-MAIT^142^^,^^143^) B cell lymphoma (CD19 targeting CAR-MAIT^142^)	neuroblastoma (GD2-targeting CAR-iNKT, NCT03294954) B cell lymphoma (CD19-targeting CAR-iNKT, NCT05487651, NCT04814004, and NCT03774654) melanoma (NCT02619058) (CSPG4-targeting CAR-iNKT^144^) solid tumors (NCT02562963) non-small cell lung cancer (NCT03198923) lymphoma (CD19-targeting CAR-iNKT^109^^,^^145^) multiple myeloma (BCMA-targeting and CD38-targeting CAR-iNKT^35^^,^^144^^,^^146^)	neuroblastoma (NCT05400603) acute myeloid leukemia (NCT03790072) solid tumors (NKG2DL-targeting γδ T, NCT04107142) hepatocellular carcinoma (NCT04518774) glioblastoma (NCT04165941) B cell malignancy (CD20-targeting CAR-γδ T, NCT04735471, and CD19-targeting CAR-γδ T, NCT02656147) CD7^+^ T cell lymphoma (CD7-targeting CAR-γδ T, NCT04702841) leukemia (CD19-targeting CAR-MAIT^111^^,^^147^)

Focusing now on MAIT cell-based immunotherapy, the adoption of innate MAIT cells still remains a novel area of CAR engineering (Figure 2A). Our past work has shown the ability to generate mesothelin-targeting CAR-MAIT cells from PBMCs via lentiviral transduction.^23^ In a three-dimensional organoid culture with M2-polarized macrophages mimicking the immunosuppressive tumor microenvironment, the cytotoxic capacity of mesothelin-targeted CAR-T cells was largely suppressed while mesothelin-targeted CAR-MAIT cells retained their potency against cancer cells, likely due to their direct recognition of tumor-associated macrophages (TAMs) through NK activating receptors and MAIT TCRs^23^ (Figures 1C and 2B). These results indicated that CAR-engineered MAIT cells show excellent targeting of TAMs in solid tumors, enhancing tumor killing through a reduction in immunosuppression from TAMs and other implicated cell types.^23^ CD19 CAR-engineered MAIT cells generated by Bohineust et al. demonstrated that these therapeutic MAIT cells could be engrafted without eliciting GvHD in preclinical immunodeficient mouse models, unlike CD19-targeted CAR-T cells.^113^

However, because MAIT cells only compose 1%–10% of the proportion of T cells within peripheral circulation, PBMC-derived MAIT cells are precluded from large-scale production, leading to limitations in the research of CAR-MAIT cells.^114^ To address the problem, genetically engineered HSCs or iPSCs could potentially be utilized to generate CAR-MAIT cells with high yield and low cost. So far, our stem cell culturing platform has been used to generate human allogeneic BCMA CAR-HSC-derived iNKT cells for off-the-shelf cancer therapy; because of their similarities as innate T cells, a modified version of this procedure could be adopted for production of HSC-derived CAR-MAIT cells. Additionally, further genetic engineering techniques could be incorporated in the development of CAR-MAIT cell products. For example, the powerful gene-editing tool, CRISPR-Cas9, could be used to knock out HLA-I and HLA-II to eliminate host T cell-mediated allorejection, or PD-1 and CTLA-4 to reduce immune checkpoint-mediated immunosuppression^115^^,^^116^^,^^117^ (Figure 2C).

## MAIT cells in GvHD amelioration

MAIT cells are restricted by MR1 and do not recognize mismatched MHC molecules and protein autoantigens; therefore, MAIT cells are not expected to induce GvHD.^19^ An *in vitro* mixed lymphocyte reaction assay indicated that MAIT cells did not cause alloresponse against multiple mismatched-donor PBMCs.^23^ A xenogeneic GvHD mouse model also showed that human MAIT cells did not expand or accumulate in immune-mediated tissue lesions during human T cell-mediated xenogeneic GvHD. These results indicate the GvHD-free safety profile of MAIT cells.^118^ In addition, multiple clinical studies have demonstrated that an increased number of MAIT cells is associated with improved overall survival and less GvHD after allogeneic hematopoietic stem cell transplantation (allo-HSCT).^27^^,^^28^^,^^29^^,^^30^^,^^31^^,^^32^^,^^33^^,^^34^

Allo-HSCT is a curative therapeutic approach for a variety of hematologic malignancies. However, its broader application is largely limited by the acute and chronic GvHD risks associated with the donor T cell-mediated alloreactive process.^119^^,^^120^^,^^121^ Cellular components of the graft, such as NK cells, B cells, T regulatory (Treg) cells, invariant natural killer T (iNKT) cells, and gamma delta T (γδ T) cells, have been shown to modulate donor T cells and reduce the risk and severity of GvHD.^27^^,^^99^^,^^122^^,^^123^^,^^124^^,^^125^^,^^126^^,^^127^^,^^128^ Recently, studies have been focused on identifying MAIT cells and their functions in gut GvHD amelioration post allo-HSCT.

In 2017, two clinical observational studies described MAIT cell reconstitution post allo-HSCT administration.^29^^,^^32^ MAIT cell reconstitution was deficient compared with other T cells, MAIT cell functionality was impaired early after allo-HSCT although restored at 24 months post allo-HSCT, and MAIT sensitivity increased toward immunosuppressive drugs (i.e., cyclosporine A and sirolimus), which might explain their impaired reconstitution.^29^^,^^32^ Interestingly, superior MAIT cell reconstitution was correlated with the increased gastrointestinal abundance of distinct bacterial species *Blautia* spp.,^32^ indicating the potential effect of gastrointestinal bacterial colonization on MAIT cell reconstitution and GvHD prevention. In 2018, another clinical study in Japan examined the MAIT and iNKT cell reconstitution post allo-HSCT, and multivariate analyses demonstrated that the absolute number of MAIT cells (<0.48/μL on day 60 post allo-HSCT), but not iNKT cells, was the only independent risk factor for grade I–IV and grade II–IV acute GvHD.^28^ High MAIT cell recovery post allo-HSCT was associated with the development of delayed-onset acute GvHD.^28^ A similar conclusion was reported by other clinical studies in China, Japan, and the United States.^27^^,^^30^^,^^31^^,^^33^ MAIT cell numbers in the graft have been shown to affect the composition of recipients’ intestinal flora, and in return the riboflavin metabolism pathway of bacterial flora could activate MAIT cells and promote generation of intestinal protective factors, altering the occurrence of gut GvHD.^27^^,^^33^

A preclinical study using syngeneic mouse models confirmed that recipient MAIT cells protected mice from acute GvHD in the colon following bone marrow transplantation.^34^ Recipient MAIT cells could generate large amounts of IL-17A, reinforce gastrointestinal tract integrity, and limit donor alloantigen presentation.^34^ Considering the higher frequency of MAIT cells in human, these cells likely represent an important population in clinical allo-HSCT. Overall, both graft and recipient MAIT cells have been shown to reduce the risk of GvHD in allo-HSCT. Thus, increasing the numbers of MAIT cells in the allograft may provide an attractive strategy for ameliorating GvHD. Thanks to their recognition of MR1 and their own safety profile, MAIT cells can also be sourced from third-party donors.

## MAIT cell-based cancer immunotherapy in combination with other treatments

Immune checkpoint inhibitors (ICIs) such as anti-PD-1 therapy have achieved significant therapeutic efficacy in a variety of malignant diseases. However, only a subset of patients show a tumor regression response to anti-PD-1, and the underlying mechanisms that contribute to the difference remain largely unclear.^129^ Early studies focusing on the tumor have shown that an increased mutational rate of tumor cells might predict the response to ICIs, although less is known about how ICIs affect the host immune response coordinated by T cells. Interestingly, several groups independently reported an observation that there is an increased frequency of MAIT cells in cancer patients at baseline and after anti-PD-1 therapy.^68^^,^^129^^,^^130^ The increased percentage of MAIT cells in the patients correlated with a favorable response to anti-PD-1 therapy in both MM and melanoma patients.^68^^,^^129^^,^^130^ In melanoma patients, it has been demonstrated that mechanistically, activated MAIT cells in anti-PD-1-responding patients showed higher expression of homing receptors such as CXCR4.^129^ The CXCR4-CXCL12 interaction is a well-established axis involved in tumor metastasis,^131^^,^^132^ and thus it is tempting to postulate that increased expression of CXCR4 on MAIT cells after anti-PD-1 treatment engenders MAIT cells’ ability to infiltrate the solid tumor microenvironment, which in turn leads to favorable outcomes. This is consistent with other evidence showing that activated MAIT cells express an array of peripheral homing receptors such as CCR6, CXCR6, and CCR9,^16^^,^^37^ and are thus intrinsically programmed to patrol peripheral tissues instead of homing to secondary lymph organs. Additionally, activated MAIT cells in patients responding to anti-PD-1 express high levels of CD69, an activation marker and indication of tissue residency.^129^ It is thus reasonable to hypothesize that activated MAIT cells are capable of infiltrating the solid tumor microenvironment and reside for a longer period of time in comparison with conventional T cells. However, further studies using *in vitro* and/or *in vivo* solid tumor microenvironment models are required to confirm this hypothesis. Nonetheless, current evidence strongly suggests a positive correlation between increased MAIT cell percentage at baseline and after anti-PD-1 therapy as a good indication for favorable outcomes. Furthermore, combination therapy using both ICIs and *ex vivo* activated MAIT cells might provide a novel strategy to boost the efficacy of ICI treatments such as anti-PD-1 therapy.

In addition to implications in ICI treatments, MAIT cells originally attracted much attention because of high expression of multidrug resistance transporter ABCB1.^2^ It has been shown that owing to this unique phenotype, MAIT cells are more resistant to chemotherapy in comparison with other T cell subtypes.^2^ Specifically, Dusseaux et al. demonstrated that after six cycles of anthracycline, a chemotherapy for breast cancer, CD4, or CD8, naive or memory conventional T cells showed significant decreased absolute cell numbers while MAIT cells did not decrease in number and stayed stable for at least 1 month after.^2^ Considering the fact that MAIT cells are found in great abundance at mucosal-associated areas such as the gut, it is not surprising that MAIT cells evolve and upregulate ABCB1 in order to efflux bacterial xenobiotics more effectively to help survive the harsh gut environment.^2^ In the context of malignant diseases, the resistance to chemotherapy suggests MAIT cells as a promising alternative cell source for development of autologous CAR-T cell therapy, since current cancer patients qualified for receiving autologous CAR-T cell therapy must have received several first-line treatments, particularly chemotherapy.

## Conclusion

Immunotherapy represents a promising new direction for cancer therapy. For example, the anti-PD-L1 ICI atezolizumab has been approved as the standard first-line therapy for treating patients with multiple cancers.^133^ Over the past decades, CAR-T cell therapy has emerged as an attractive immunotherapy approach for the treatment of hematologic malignancies and solid tumors. However, current CAR-T cell therapy has significant limitations: its efficacy needs to be improved and, importantly, it is an autologous treatment, making the therapy extremely costly and difficult to deliver to all cancer patients in need. To fully harness the potential of CAR-directed cell therapy, the development of a potent off-the-shelf cell therapy is necessary.^92^^,^^93^ By virtue of the powerful antitumor capacity, long-term persistence, high safety profile, and availability of genetic engineering, MAIT and CAR-MAIT cells have been explored as promising candidates for cancer immunotherapy. Given the increased numbers of MAIT cells in mucosal-associated peripheral tissues such as lung, gastrointestinal tract, colon, and cervix,^58^^,^^61^^,^^62^^,^^63^^,^^64^^,^^66^ it is likely that cancers located in these types of tissues may be more amenable to MAIT cell-based therapy.

Like the conventional T/CAR-T cell products, the proposed MAIT/CAR-MAIT cell products confront the same limitations that need further improvement. For example, CAR-MAIT cells are expected to be potent immunomodulatory and cytotoxic immune cells which may induce the risk of CRS and neurotoxicity side effects.^134^^,^^135^^,^^136^ A dose-escalation and regimen study at phase I clinical trials will help to define an optimal therapeutic protocol. The fast-developing autologous CAR-T cell therapy also has accumulated valuable clinical experiences handling these side effects, such as giving patients anti-IL-6 treatment.^137^ The sr39TK suicide gene incorporated in the CAR-MAIT cell product can also serve as a “kill switch” in the case of severe adverse effects.^35^^,^^101^ Another major concern of allogeneic MAIT cell products is their possible allorejection by the host immune cells, which may limit their therapeutic efficacy. Ablation of HLA-I and HLA-II molecule expressions on MAIT cells could render these cells resistant to the host CD8^+^ and CD4^+^ T cell-mediated killing, respectively.^35^^,^^138^ Ablation of HLA-I expression on MAIT cells may make them targets of host NK cells, which can be addressed by delivering into MAIT cells an NK-inhibitory gene such as HLA-E or HLA-G.^35^^,^^139^^,^^140^

Some powerful treatment strategies for future MAIT cell-based therapy are aimed at establishing iPSCs that can produce unlimited numbers of MAIT cells with enhanced immune functions. Another option is to incorporate the MAIT cell vaccines containing tumor mRNA and 5-OP-RU/MR1; the CAR-T vaccines have been shown to trigger massive CAR-T cell expansion and enhanced antitumor efficacy in multiple tumor models.^141^ In addition, the combination of MAIT cell-based therapy with other treatments (e.g., chemotherapy or checkpoint blockade) could be a good option for cancer patients who are resistant to current immunotherapies.
